# ﻿Two new species of the genus *Glaucocharis* (Lepidoptera, Crambidae) from China

**DOI:** 10.3897/zookeys.1260.152038

**Published:** 2025-11-13

**Authors:** Xinxin He, Chao Jiang, Qidi Zhu, Weichun Li

**Affiliations:** 1 College of Agronomy, Jiangxi Agricultural University, Nanchang 330045, China; 2 State Key Laboratory for Quality Ensurance and Sustainable Use of Dao-di Herbs, National Resource Center for Chinese Materia Medica, China Academy of Chinese Medical Sciences, Beijing 100700, China; 3 Jiangxi Provincial Key Laboratory of Conservation Biology, Jiangxi Agricultural University, Nanchang 330045, China

**Keywords:** China, Crambinae, grass moths, morphology, new species, taxonomy, Xizang

## Abstract

Two new species of the genus *Glaucocharis*, *Glaucocharis weii* Li & He, **sp. nov.**, and *G. pangda* Li & He, **sp. nov.**, encountered in the primaeval forests of southern Xizang of China, are described. Images illustrating the adult morphology of the new species are provided.

## ﻿Introduction

Meyrick (1938) established the genus *Glaucocharis* (Crambidae, Crambinae) and assigned *Glaucocharis stella* Meyrick, 1938 as the type species. The genus includes 163 species with Oriental, Palearctic, African and Australian distributions (Nuss et al. 2003–2025; Léger 2024; Matsui et al. 2025). Prior to this study, sixty-two species were known from China (Wang and Sung 1983; Wang et al. 1988; Chen et al. 2002; Li and Li 2012; Li 2018). The present study aims to add two new species to science based on specimens collected in the primaeval forests of southern Xizang, China.

## ﻿Material and methods

Specimens were collected at night using a 250W mercury-vapor lamp and killed with ammonium hydroxide. The specimens studied are deposited in the Insect Museum, Jiangxi Agricultural University, Nanchang, China (**JXAUM**) and the National Zoological Museum of China, Institute of Zoology, Chinese Academy of Sciences, Beijing, China (**NZMCAS**).

Terminology for morphological structures follows Li and Li (2012). Photographs of adults were taken using a Zeiss AxioCam ICc 5 camera attached to a Zeiss Stereo Discovery V12 microscope. Illustrations of the genitalia were prepared with an Optec DV E3 630 digital camera connected to an Optec BK6000 microscope.

## ﻿Results

### ﻿ Glaucocharis weii

Taxon classificationAnimaliaLepidopteraCrambidae

Li & He sp. nov.

257ACB99-C29E-54F0-9ECF-2494482F615E

https://zoobank.org/A6B11855-FA34-4BF4-89CF-B3E9B6829CBF

Fig. 1

#### Material examined.

***Holotype***: China • ♂, Xizang Autonomous Region, Xigaze City, Yadong County, Pangda Village (27°21'N, 88°59'E), alt. 2382 m, 23 July 2024, W Li and X He et al. leg. (JXAUM). ***Paratypes***: • 5 ♀♀, same data as holotype (JXAUM); • 1 ♂, 1 ♀, same data as holotype (NZMCAS); • 5 ♂♂, 6 ♀♀, Xizang Autonomous Region, Shannan City, Cuona County, Lebugou (27°49'N, 91°44'E), alt. 2629 m, 16 July 2024, W Li and X He et al. leg. (JXAUM).

**Figure 1.**
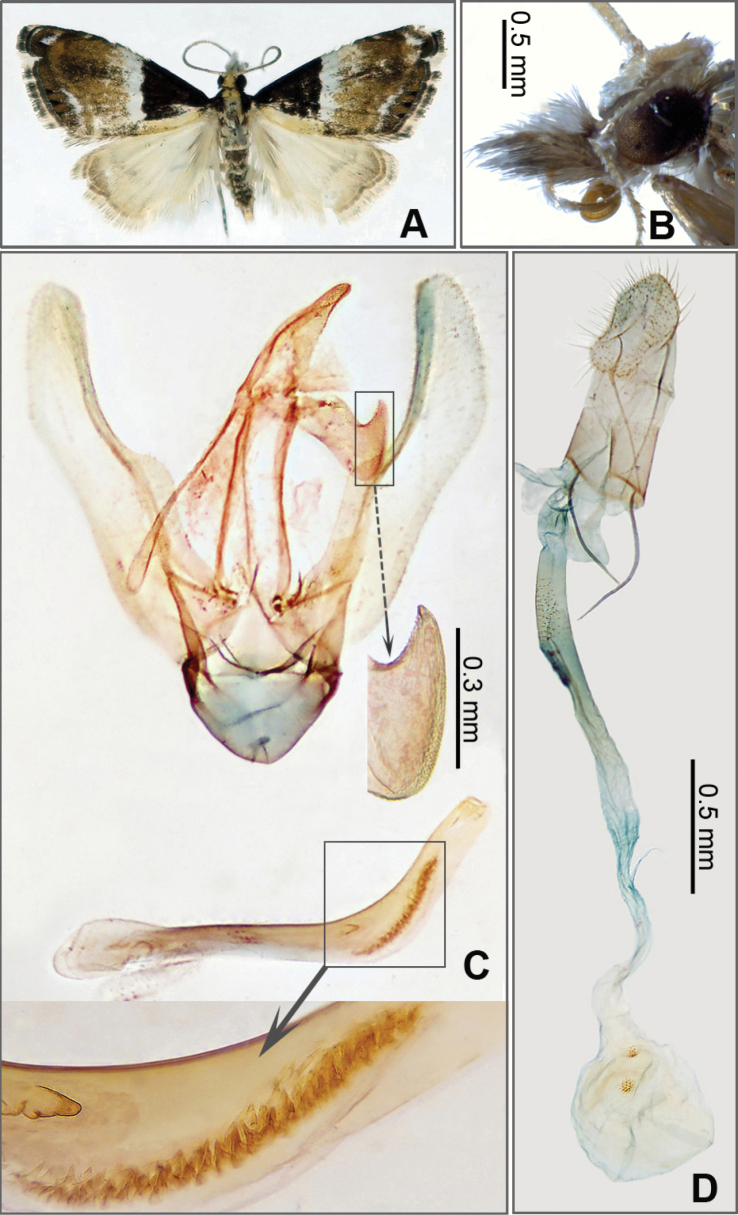
*Glaucocharis weii* Li & He, sp. nov. A. Adult in dorsal view, female, paratype (JXAUM); B. Head in lateral view, male, holotype (JXAUM); C. Male genitalia, holotype, genitalia slide no. HX24075; D. Female genitalia, paratype, genitalia slide no. HX24080.

#### Diagnosis.

In male genitalia, the gnathos distally with a triangular projection on dorsal margin and a protuberance on ventral margin, and the cornuti consisting in a cone-like process and a row of tiny spines. In female genitalis, the ductus posterior one-third slightly sclerotized, and the corpus bears double and rounded signa. This species is similar to *G. taphrophracta* (Meyrick, 1934) in the triangular projection on the dorsal margin of the uncus in male genitalia (Fig. 1C versus Wang et al. 1988: fig. 87). It can be distinguished by the concave costa, and cornuti consisting in a cone-like process and a row of tiny spines of various sizes (Fig. 1C). Whereas in *G. taphrophracta*, the costa is almost straight and the vesica of the phallus bears two thin and long cornuti (Wang et al. 1988: 375, fig. 87).

#### Description.

***Adult habitus*** (Fig. 1A, B). Forewing length 6.0–7.0 mm. Frons and vertex white. Labial palpus first and second segments uplifted, third segment projecting forward; outer side surface pale yellow mixed with brown except for first segment white at base. Maxillary palpus white mixed with brown. Antenna alternately pale brown and yellowish brown on dorsal surface. Patagium pale yellow. Tegula blackish brown. Thorax pale yellow. Forewing densely covered with black scales on inner side of antemedian line, pale yellow along dorsum; antemedian line white, inner margin wavy; reniform stigma unrecognized; postmedian line yellowish brown, outcurved at costal one-third; apex black, with white spot; termen black mixed with yellowish brown, with three black marginal spots; fringe blackish brown mixed with yellowish white. Hindwing yellowish white; postmedian line pale brown; apex scattered with pale brown scales; fringe yellowish white mixed with pale brown. Legs yellowish white; tarsi alternately black and white. Abdomen with first and second segments grey, genital segment yellowish white, other segments alternately brown with yellowish white.

***Male genitalia*** (Fig. 1C). Uncus base broad, narrowed towards blunt apex. Gnathos slightly longer than uncus, distal apex with triangular projection on dorsal margin, and protuberate on ventral margin. Tegumen approximately twice as long as gnathos. Valva with basal half broader than distal half, gently concave near middle of ventral margin, apex round, costa gently concave. Saccus well-developed. Juxta elliptic. Phallus nearly as long as valva, cornuti consisting in cone-like process and a row of tiny spines of various sizes.

***Female genitalia*** (Fig. 1D). Papillae anales about half as long as apophyses posteriores. Apophyses anteriores thin and long, nearly as long as apophyses posteriores. Ductus bursae thin and long, posterior one-third slightly sclerotized. Ductus seminalis arising from approximately anterior one-third of ductus bursae. Corpus bursae rounded; signa double and rounded.

#### Etymology.

This species is named in honour of Academician Fuwen Wei, a renowned conservation biologist who has made profound contributions to biodiversity, zoological evolution, and conservation biology.

### ﻿ Glaucocharis pangda

Taxon classificationAnimaliaLepidopteraCrambidae

Li & He sp. nov.

BEB521A6-2807-582C-8DD8-D7F4D509EA25

https://zoobank.org/752D71E2-B3D8-45FD-9E22-85185B4ED9AE

Fig. 2

#### Material examined.

***Holotype***: China • ♂, Xizang Autonomous Region, Xigaze City, Yadong County, Pangda Village (27°19'N, 89°00'E), alt. 2192 m, 24 July 2024, W Li and X He et al. leg. (JXAUM). ***Paratypes***: • 7 ♀♀, same data as holotype (JXAUM); • 2 ♀♀, same data as holotype except for alt. 2382 m, 23 July 2024 (NZMCAS).

**Figure 2.**
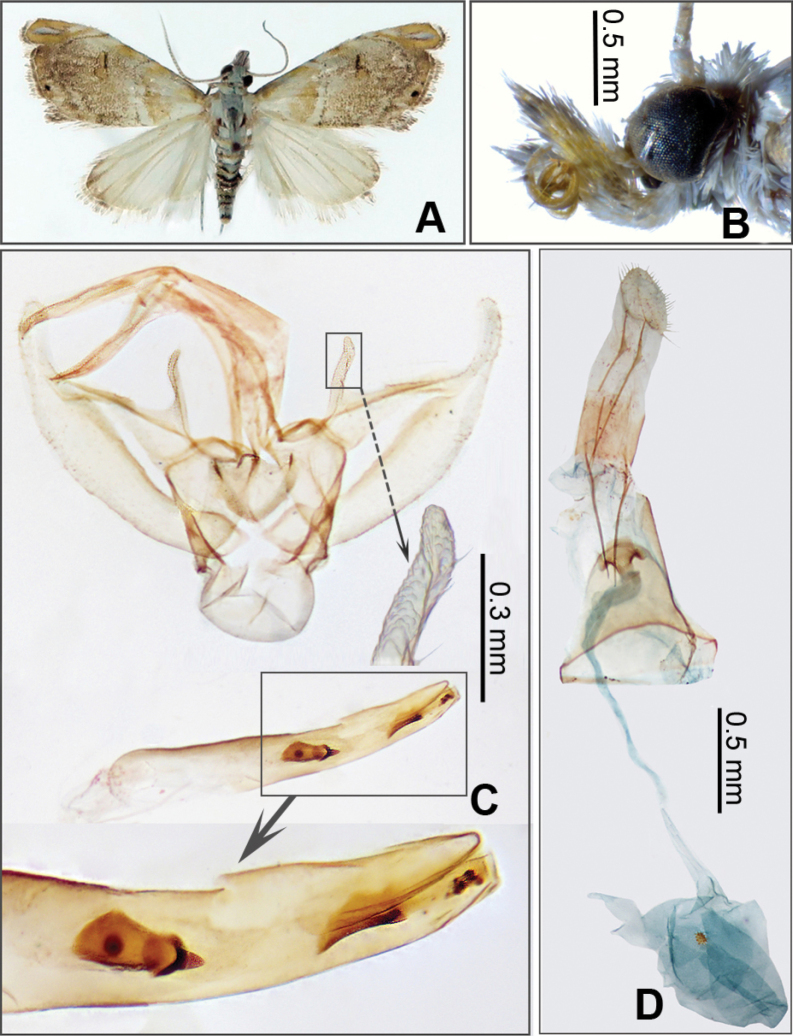
*Glaucocharis pangda* Li & He, sp. nov. A. Adult in dorsal view, female, paratype (JXAUM); B. Head in lateral view, male, holotype (JXAUM); C. Male genitalia, holotype, genitalia slide no. HX24084; D. Female genitalia, paratype, genitalia slide no. HX24093.

#### Diagnosis.

In male genitalia, the costa has a densely granulate projection near base, and the phallus contains three sclerotized plates and a denticle. In female genitalia, the antrum is shield-shaped. This species is similar to *G. apicudentella* Song & Chen, 2001 by having a thin and long projection near the costal base of the valva in male genitalia (Fig. 2C versus Chen et al. 2001: fig. 4). It can be distinguished by the valva with a round apex, the juxta distally with two short lateral prongs, and the phallus with a large conical denticle in the centre of the uneverted vesica, an elongate rod-like structure further towards the posterior end of the phallus, and a small granular patch near the posterior opening of the phallus (Fig. 2C). Whereas in *G. apicudentella*, the valva ends with two sclerotized thorns, the juxta distally bears two long lateral prongs, and the vesica of the phallus lacks a cornutus (Chen et al. 2001: 173, fig. 4).

#### Description.

***Adult habitus*** (Fig. 2A, B). Forewing length 6.0–6.5 mm. Frons and vertex white. Labial palpus projecting upward; outer side surface yellow mixed with brown except for first segment white at base. Maxillary palpus pale yellow mixed with white. Antenna alternately pale brown and yellowish white on dorsal surface. Patagium and tegula white mixed with pale yellow. Thorax white. Forewing scattered with white and pale yellow scales; antemedian line white, angled outwards near costa, then inclined inwards; reniform stigma 8-shaped, pale yellow, with straight black bar on posterior margin; postmedian line white, outcurved at costal one-third; apex pale yellow, with slender white streak margined with black; termen pale brown mixed with pale yellow, bearing two black marginal spots: one at two-thirds and a smaller one in the middle; fringe pale brown mixed with yellowish white. Hindwing yellowish white; apex scattered with pale brown scales; fringe white mixed with pale brown. Legs yellowish white. Abdomen with first to third segments alternately white and pale yellow, other segments alternately blackish brown and white on dorsal surface, yellowish white on ventral surface.

***Male genitalia*** (Fig. 2C). Uncus thin and long, apex pointed. Gnathos slightly shorter than uncus, apex pointed. Tegumen a bit longer than gnathos. Valva base broad, gently narrowed towards distal part; distal one-fourth thin and long, apex round, costa with thin and long, densely granulate projection near base. Saccus elliptic. Juxta base narrow, broadened towards distal apex, distally with two short lateral prongs. Phallus almost as long as valva, vesica bearing a large conical denticle in centre of uneverted vesica, an elongate rod-like structure further towards posterior end, and a small granular patch near posterior opening.

***Female genitalia*** (Fig. 2D). Papillae anales approximately one-third as long as apophyses posteriores. Apophyses anteriores thin and long, slightly shorter than apophyses posteriores. Antrum shield-shaped. Ductus bursae thin and long. Corpus bursae rounded; single signum circular.

#### Etymology.

This species is named after Pangda Village, where the type specimens were collected.

## Supplementary Material

XML Treatment for Glaucocharis weii

XML Treatment for Glaucocharis pangda

## ﻿Additional information

### Use of AI

No use of AI was reported.

### Author ORCIDs

Chao Jiang https://orcid.org/0000-0003-1841-1169

Qidi Zhu https://orcid.org/0000-0001-8187-1286

Weichun Li https://orcid.org/0000-0003-0154-861X

### Data availability

All of the data that support the findings of this study are available in the main text.
